# From Cellular Infiltration Assessment to a Functional Gene Set-Based Prognostic Model for Breast Cancer

**DOI:** 10.3389/fimmu.2021.751530

**Published:** 2021-10-04

**Authors:** Huamei Li, Yiting Huang, Amit Sharma, Wenglong Ming, Kun Luo, Zhongze Gu, Xiao Sun, Hongde Liu

**Affiliations:** ^1^ State Key Laboratory of Bioelectronics, School of Biological Science & Medical Engineering, Southeast University, Nanjing, China; ^2^ Department of Neurosurgery, Center for Integrated Oncology (CIO), University Hospital Bonn, Bonn, Germany; ^3^ Department of Integrated Oncology, Center for Integrated Oncology (CIO), University Hospital Bonn, Bonn, Germany; ^4^ Department of Neurosurgery, Xinjiang Evidence-Based Medicine Research Institute, First Affiliated Hospital of Xinjiang Medical University, Urumqi, China

**Keywords:** breast cancer, specific gene expression profile, cellular infiltration, prognosis, risk score, immunotherapy, cancer heterogeneity

## Abstract

**Background:**

Cancer heterogeneity is a major challenge in clinical practice, and to some extent, the varying combinations of different cell types and their cross-talk with tumor cells that modulate the tumor microenvironment (TME) are thought to be responsible. Despite recent methodological advances in cancer, a reliable and robust model that could effectively investigate heterogeneity with direct prognostic/diagnostic clinical application remained elusive.

**Results:**

To investigate cancer heterogeneity, we took advantage of single-cell transcriptome data and constructed the first indication- and cell type-specific reference gene expression profile (RGEP) for breast cancer (BC) that can accurately predict the cellular infiltration. By utilizing the BC-specific RGEP combined with a proven deconvolution model (LinDeconSeq), we were able to determine the intrinsic gene expression of 15 cell types in BC tissues. Besides identifying significant differences in cellular proportions between molecular subtypes, we also evaluated the varying degree of immune cell infiltration (basal-like subtype: highest; Her2 subtype: lowest) across all available TCGA-BRCA cohorts. By converting the cellular proportions into functional gene sets, we further developed a 24 functional gene set-based prognostic model that can effectively discriminate the overall survival (*P* = 5.9 × 10^−33^, *n* = 1091, TCGA-BRCA cohort) and therapeutic response (chemotherapy and immunotherapy) (*P* = 6.5 × 10^−3^, *n* = 348, IMvigor210 cohort) in the tumor patients.

**Conclusions:**

Herein, we have developed a highly reliable BC-RGEP that adequately annotates different cell types and estimates the cellular infiltration. Of importance, the functional gene set-based prognostic model that we have introduced here showed a great ability to screen patients based on their therapeutic response. On a broader perspective, we provide a perspective to generate similar models in other cancer types to identify shared factors that drives cancer heterogeneity.

## 1 Introduction

Cancer biology has now reached a point where it is well understood that cancer cells interact with their microenvironment, which ultimately determines whether it will respond to treatment, develop resistance, recur or metastasize. Therefore, it is a must to recapitulate the prevailing information on various cancer models to draw some stringent conclusions connecting the common/shared factors involved in the tumor microenvironment (TME). Considering this, herein, we focused on breast cancer (BC), which is the most common invasive disease and the leading cause of cancer death in women worldwide (1). Despite the partial success of conventional therapies (surgery, chemotherapy, radiotherapy, and targeted therapy) and other ongoing *therapeutic* advances (immunotherapy), it remains a concern why some patients eventually develop metastases and others respond poorly to treatment. Currently, the assessment of the prognostic and predictive significance of tumor-infiltrating lymphocytes (TILs) in BC is gaining quite a momentum (2, 3). Since TILs comprise a heterogeneous population of cells with different physiological/pathological effects in the tumor microenvironment (TME), therefore, new emerging technologies (e.g., single-cell RNA sequencing: scRNA-seq) have gained an advantage in resolving their functional interpretation in BC (4).

While the accuracy of predicting the cellular composition is an imperative factor to understand the heterogeneity associated with TME (5–7), the defined analysis of bulk datasets using a robust deconvolution strategy is also an considerably important parameter (5, 6, 8–11). To some extent, reference gene expression profiling (RGEP) has proven to be successful in this context, as evident from studies using RGEP either by, 1) directly using scRNA-seq data, such as the head and neck squamous cell carcinoma RGEP (called HNSCC-RGEP hereafter), or 2) using sorted bulk gene expression datasets, such as LM22 (9), ImmunoStates (12) and ABIS (13). Given that the reliability of RGEPs depends on disease-specific gene expression patterns, disease status/stage, and diversity within the tissue cell population, it is necessary to consider multiple parameters ranging from direct health/disease status to complex indicators (tissue- and disease-specific) (12, 14, 15). Interestingly in BC, a few studies have provided prognostic models based primarily on the cellular proportions (16, 17). However, when applying non-specific RGEPs to predict the cellular compositions of patients, the technical bias can be expected, therefore, the reliability of the prognostic models will come under concern. Of interest, one study suggested that the pathway-based prognostic models performed systematically better than gene-based models and proposed that by including the clinical information, the prognostic prediction of such models can be further enhanced (18).

Considering all these facts, herein, we aimed to establish BC-specific RGEP by using scRNA-seq datasets, as an initial perspective that can be used in the future to generate similar models in other cancer types to identify common factors driving cancer heterogeneity. Our work primarily focused on previously reported 15 cell types (including fibroblasts, malignant cells, and 13 immune cell types) of BC patients (19), combined with our recently published deconvolution method (LinDeconSeq) (8) and comprehensive comparisons with the preexisting RGEPs. As an extended application, we also developed 24 functional gene sets (biological processes and signaling pathways) to correlate infiltration of prognosis-related cell types, in order to obtain a robust prognostic value (risk groups, therapeutic regimens) from BC cohorts.

## 2 Materials And Methods

### 2.1 Datasets

The BC-related datasets used in this study were retrieved from the Gene Expression Omnibus (GEO) (accession numbers: GSE114725, GSE75688, GSE5462, GSE18728, GSE41998, GSE37946, GSE25066). Similarly, the gene expression and phenotype data (an open access level 3 gene expression matrix data) of TCGA-BRCA and other 32 cancer types were obtained from The Cancer Genome Atlas Project (TCGA). Additionally, three BC datasets (Caldas, Chin, and Yao), along with their phenotype details were retrieved from the GDC Xena Hub (https://xenabrowser.net/datapages/). The Molecular Taxonomy of Breast Cancer International Consortium (METABRIC) datasets were accessed from the European Genome-Phenome Archive (EGA) using accession number EGAS00000000083. Other gene expression datasets such as NKI, Mainz, Transbig, UNT, and UPP were obtained from the R Bioconductor packages, *breastCancerNKI*, *breastCancerMAINZ*, *breastCancerTRANSBIG*, *breastCancerUNT* and *breastCancerUPP*, respectively. The scRNA-seq datasets of BC (Bassez et al.) that received anti-PD-1 were retrieved upon request from the website https://lambrechtslab.sites.vib.be/en/single-cell (20). In the absence of any published datasets of BC patients receiving immunotherapy, we utilized a urothelial cancer dataset that received anti-PD-L1 therapy (IMvigor210), and was downloaded from the R package *IMvigor210CoreBiologies* (version 1.0.0) (21). The details about all these datasets were given in **Supplementary Table S1**. To mention, all these samples were not pre-screened, but only tumor (normal samples were excluded) samples were included in the prognostic analysis. In addition, our newly establish BC-specific RGEP was compared with the external RGEPs including LM22 (9), Yu et al.’s (HNSCC-RGEP) (14), ABIS (13), immunoStates (12), which were obtained from the attachments or links given in these articles.

### 2.2 Methods

#### 2.2.1 Normalization of Bulk Gene Expression Data for BC Cohorts

Particularly for microarray datasets (from NCBI-GEO), both background correction and quantile normalization were performed using the Robust Multiarray Averaging (RMA) method (22). In case of bulk RNA-Seq and scRNA-Seq datasets, the gene expression profiles were normalized as counts per million (CPM) quantifications and were then subjected to natural-log transformation.

#### 2.2.2 Construction of BC-Specific RGEP

BC-specific RGEP is tissue and disease-specific reference matrix derived from breast tumor scRNA-seq data, where the rows represent genes and columns are cell types. It should be mentioned that each entry represents the average expression of the gene within that cell type. The details on the construction of the BC-specific RGEP have been provided below.

##### 2.2.2.1 Pre-Processing and Clustering of BC scRNA-Seq Data

**Figure 1:**
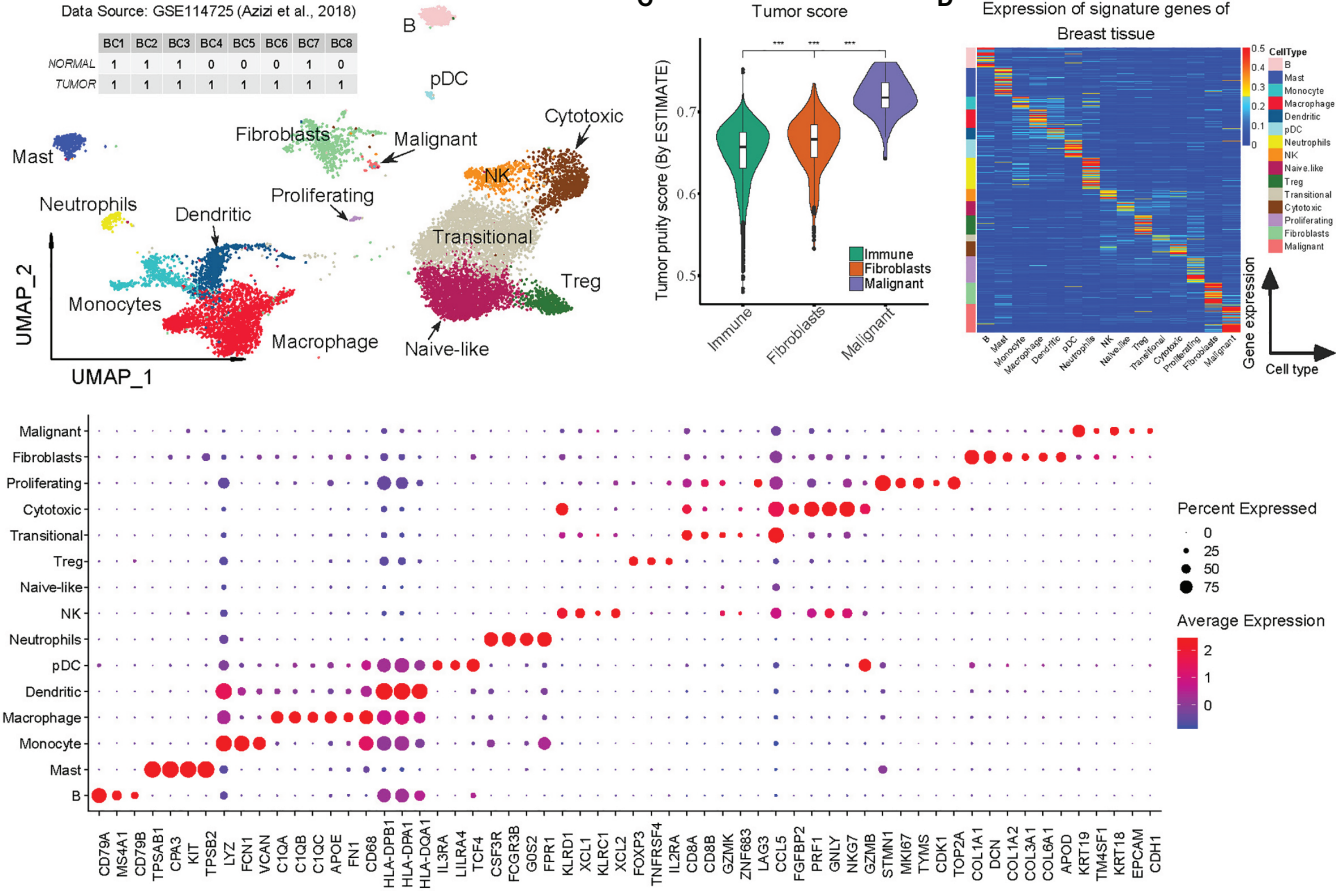
Construction of BC-specific RGEP for cell type deconvolution using scRNA-seq data. **(A)** Two-dimensional UMAP plot of 12132 single cells from 8 breast cancer patients. Each point represents one single cell, colored according to cell cluster. **(B)** Dot-plot showed the expression of the selected DEGs in each cell cluster. **(C)** Tumor score was inferred from the stromal and immune signature using ESTIMATE algorithm (25). Each box shows the median and interquartile range (IQR 25th–75th percentiles), whiskers indicate the highest and lowest value within 1.5 times the IQR and outliers are marked as dots. *P-*value, Student’s *t*-test (***p < 0.001). **(D)** The expression of the signature matrix of breast tissue, the expression was row-normalized (normalize each expression value by the sum over the row) across cell types. The upper bound of the color bar is 1.

Raw UMI count matrix data of scRNA-seq obtained from eight BC patients (GEO ID, GSE114725) (19) and was analyzed using Seurat (version 4.0.1) (23). The cells with <200 or >3,000 expressed genes and those with <500 or >10,000 UMIs were discarded (**Supplementary Figure 1A**, 22,970 cells were retained). The raw UMI counts were then log-normalized with a scale of 10,000, and highly variable genes were identified using the *vst* method. In order to eliminate batch effects across samples and biological effects among normal and tumor states, the first 30 principal components tool was extracted using the integration tool Harmony (24). Cells were then clustered using the *FindCluster* function and *resolution* = 0.5. We found that both clusters 11 and 17 had highly outlier distributions of expressed genes and UMI counts and filtered out (**Supplementary Figure 2B**). Following these processing steps, there remained 12,132 cells clustered into 17 groups for the cell type annotations. We manually annotated the cell types by comparing the canonical markers with the differential expression genes identified by the *FindAllMarkers* method with *logfc.threshold* = 0.5 and *min.pct* = 0.1 (**Figure 1B** and **Supplementary Table S2**).

##### 2.2.2.2 Selection of Cell Type-Specific Genes (Also Called Signature Genes)

An accurate deconvolution requires the selection of cell type-specific genes (i.e. the signature genes) whose expression levels must be informative enough to distinguish the cell types throughout the sample (12, 15). Hence, we combined four gene sets [750 DEGs identified by the *FindAllMarkers* (Seurat, version 4.0. 1) method at *logfc. threshold* = 0.5 and *min.pct* = 0.1 (23), 547 genes identified from LM22 (9), 317 genes from immunoStates (12), 635 genes from the Tirosh I et al. study (26)] to construct a reliable BC-specific RGEP. Following removal of duplicates, 1506 unique genes were used as signature genes for the BC-specific RGEP.

#### 2.2.3 Construction of Simulated and Realistic “Bulk” Gene Expression Data

The simulated bulk gene expression samples were generated from a random proportion of 15 cell types (provided in BC-specific RGEP) using the Dirichlet distribution, followed by replaced sampling from the GSE114725 (19) scRNA-seq dataset based on the random proportions. This resulted in a total of 100 samples, with each sample containing 10,000 cells. To validate the BC-specific RGEP, two scRNA-seq datasets (Bassez et al., and GSE75688) (20, 27), where the cellular proportions for each patient sample were known in advance, were used as realistic bulk gene expression data (by aggregating reads from all cell barcodes for each patient sample).

#### 2.2.4 Deconvolution and Estimation Quality Assessment

To evaluate the performance of BC-specific RGEP, we used LinDeconSeq, a deconvolution toolkit that we recently developed using weighted robust linear regression (8). The accuracy of deconvolution was assessed by the Pearson correlation coefficient *r* and the root-mean-square error (RMSE), mainly calculated from the true and estimated cellular proportions across all the patients.

#### 2.2.5 Functional Gene Set-Based Prognostic Model

To accurately predict the prognosis and therapeutic benefits of BC patients, we proposed a functional gene set-based prognostic model, the construction of which consisted of three main steps: converting gene expression into activation scores of functional gene sets, identifying functional gene sets significantly associated with cellular proportions, and establishing the prognostic model based on the identified functional gene sets in the previous step. The details of each step were as follows.

##### 2.2.5.1 Calculation of Activation Score Using Gene Set Variation Analysis (GSVA) Tool

To assess the activation of 9,321 functional gene sets [the union of H (hallmark gene sets), C2 (curated gene sets) and C5 (ontology gene sets) from MSigDB (28)] for each patient in BC cohort, we exclusively used a nonparametric and unsupervised software algorithm called *GSVA* (29) in the R package with the microarray mode.

##### 2.2.5.2 Identification of Functional Gene Sets Significantly Associated With Cellular Proportions

After estimating the activation scores (called “GSVA score” hereafter) of functional gene sets for each BC patient, we further calculated the correlations between the proportions of 11 cell types estimated from the TCGA-BRCA cohort and the GSVA scores, and subsequently performed Fisher Z-transformations by equation 1.


(1) $${\bar{z}}_{g}=\frac{1}{2}\sum_{C\in cellTypes}\ln\left(\frac{1+r_{gC}}{1-r_{gC}}\right)$$

Where *r_gC_* is the Pearson’s correlation of gene set *g* with cell type *C*. Then standardize the Fisher-transformed correlations by their median and median absolute deviation (MAD):


(2) $$S_{g}=\frac{{\bar{z}}_{g}-median(z)}{1.4826\times MAD(z)}$$

*P*-values were then calculated for *Sg* using the standard normal distribution, and functions with *P*-values less than 0.01 were considered significantly associated with cellular proportions.

##### 2.2.5.3 Establishing the Prognostic Model (Proportion-Based Model Also Apply)

Based on the identified functional gene sets mentioned above, LASSO-Cox and multivariate Cox regression methods were applied to identify the most effective functional gene sets (or cell types for the proportion-based prognostic model) to build a prognostic model. LASSO-penalized Cox regression was used to filter out less relevant factors. Multivariate Cox regression analysis was applied to optimize the model. An optimal risk assessment model was constructed utilizing the regression coefficients derived from Cox regression multivariate analysis by multiplying the GSVA score (or cellular proportion for the proportion-based prognostic model) of each function.

#### 2.2.6 Kaplan-Meier Survival Curve

The prognostic model was designed to provide a risk score corresponding to each patient. Kaplan-Meier (KM) survival analysis was performed in combination log-rank test to determine whether the high- and low-risk groups identified by the *surv_cutpoint* function [implemented in the R package *survminer* (version 0.4.2)] exhibit significantly different survival patterns or not. In addition, the log-rank test determined whether the estimated survival curves were the same for each group, and in the case that the *P*-value is less than 0.05, the survival curves were statistically different.

#### 2.2.7 Differentially Expressed Genes (DEGs) Associating With the Prognostic Risk Groups

To identify DEGs between high- and low-risk groups, we corrected for the batch effects between BC cohorts using Combat (30). These DEGs were then determined using the R package *Limma* (31), and were further defined at the threshold of |*log_2_FC*| > 0.1 and Benjamini-Hochberg adjusted *P-value* ≤ 0.01, primarily to calculate the statistically significant differences in gene expression.

#### 2.2.8 Functional Enrichment Analysis

Gene annotation enrichment analysis for DEGs between high- and low-risk groups was performed using the R package *clusterProfiler* (32). Gene Ontology (GO) terms and KEGG pathways were considered statistically significant according to the Benjamini-Hochberg (33), adjusted *P-value* < 0.01.

#### 2.2.9 Immunoreactivity Characterization

Immunophenoscore (IPS) uses a number of markers of immune response or immune toleration to quantify four different immune-phenotypes in a tumor sample, including antigen presentation, effector cells, suppressor cells, and checkpoint markers. A z-score summarizing these four categories is generated, with a higher z-score of IPS indicating a more immunogenic sample (34, 35). In addition, scores of exhaustion, cell cycle, and activation gene sets were calculated by GSVA toolkit (see **Supplementary Table S5**) (29).

#### 2.2.10 Classification Analysis

To distinguish ER-positive/negative subtypes, a support vector machine (SVM) classifier was applied to 80% of the samples in the TCGA-BRCA cohort using parameters from five-fold cross-validation with standard parameters (using R package *e1071*). The remaining samples were used for classifier testing. A random forest model with *ntree* = 2000 (R package *randomForest*) was used to distinguish high- and low-risk BC patients. To mention, here the training set used 80% of the ten BC cohort samples, while the remaining 20% was used for testing (**Supplementary Table S1**). The receiver operating characteristic (ROC) curve was used to assess the classification performance of the model, and the area under the curve (AUC) was calculated using the *pROC* package (36).

#### 2.2.11 Code Availability

The custom codes are available from the corresponding authors upon request.

## 3 Result

### 3.1 Construction of the Reliable and Robust BC-Specific RGEP

As mentioned earlier, both indication-specific (tissue and disease type) and cell type-specific reference from scRNA-seq data is a key to deconvolute the cellular composition (15). We therefore initially obtained a total of 22,970 cells (after initial quality control) from the normal and cancerous tissues of eight BC patients (GEO accession number: GSE114725) (19) (**Supplementary Figures 1A, B** and **Supplementary Table S1**). As previously suggested (12), we applied the integration toolkit Harmony (24) to simultaneously eliminate technical bias caused by the batch effects and/or biological effects in the normal and tumor samples. As a result, 19 different cell clusters were identified, of which two (clusters 11 and 17) showed excessive outliers in the distribution of expressed genes and UMI counts (possibly enriched with the duplicate cells), hence, were excluded from the analysis (**Supplementary Figures 1B, C**). The integrated visualization revealed an extensive mixing of shared cell populations among patients and between the normal and tumor states, indicating that biases were significantly reduced (**Supplementary Figure 1D**).

On the basis of canonical cell markers, we identified 15 cell types for the clusters, including BC malignant cells (mainly characterized by the expression of *KRT19*, *KRT18*, *CDH1*, *EPCAM*), fibroblasts (*COL1A1*, *COL1A2*, *DCN*), proliferating T cells (*STMN1*, *MKI67*), cytotoxic T cells (*FGFBP2*, *NKG7*, *PRF1*), Transitional T (*CD8A*, *CD8B*, *GZMK*, *CCL5*), Treg (*FOXP3*, *TNFRSF4*), Naive-like T cells (*IL7R*, *TCF7*), NK cells (*KLRD1*, *KLRC1*), neutrophils (*CSF3R*, *FCGR3B*, *G0S2*), pDC (*IL3RA*, *LILRA4*), dendritic cells (*HLA-DPB1*, *HLA-DPA1*), macrophages (*C1QA*, *C1QB*, *FN1*), monocytes (*LYZ*, *FCN1*, *VCAN*), mast cells (*TPSAB1*, *CPA3*), and B cells (*CD79A*, *MS4A1*, *CD79B*) (**Figures 1A, B** and **Table S2**). The high correlations (*r* > 0.8) of the aggregated expression profiles between the immune cell types that we observed were consistent with one previous study (19), confirming the reliability of our annotated cell types. It should be mentioned that the study we used for comparison also included malignant cells and fibroblasts, so we applied ESTIMATE (25) to analyze the tumor purity scores and found that malignant cells had the highest tumor purity, followed by the fibroblasts, while immune cells had the lowest, which is consistent with the findings by Chung et al. (27) (**Figure 1C**). To this end, the results clearly support the reliability and adequacy of our annotated cell types for BC scRNA-Seq data.

After the cluster annotation and validation, approximately 12,132 high-quality cells were retained of which transitional T-cells were predominant while few other cell types (proliferating T cells, pDCs, and malignant cells) accounted for a very small proportion (**Supplementary Figure 1F**). In order to create a reliable and robust BC-specific RGEP for deconvolution, we averaged the gene expression within each cell type, and only the cell type-specific genes (signature genes) were retained. In the end, a specific RGEP with 1506 genes and 15 cell types was determined for the BC. The average expression levels of the signature genes were found to be specific for each cell type (**Figure 1D** and **Supplementary Table S3**). Notably, we also specified the expression of highly correlated genes (due to close cell lineages), primarily to optimize the covariance in the deconvolution model (**Supplementary Figure 1G**).

### 3.2 BC-Specific RGEP Outperformed Non-BC-Specific RGEPs in Capturing the Intrinsic Heterogeneity of BC Cohorts

**Figure 2:**
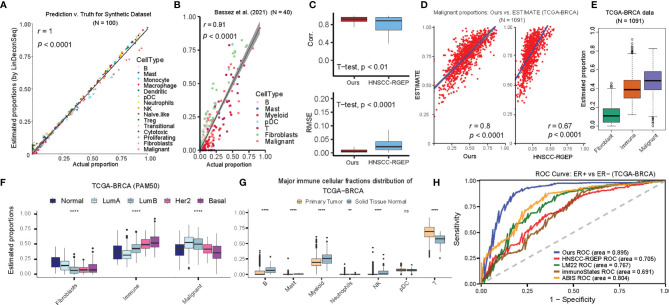
Accuracy of cellular proportions estimated using BC-specific RGEP. **(A)** Scatter-plot of the estimated and true cellular proportions for the 100 simulated bulk breast tumor samples. Each dot represents one sample and *r* denotes the Pearson’s correlation coefficient. *P*-value, Student’s *t*-test. **(B)** Scatter-plot of the estimated and true cell proportions for the Bassez et al.’s scRNA-seq breast cancer data (20). Each dot represents one patient and *r* denotes the Pearson’s correlation coefficient. *P* value, Student’s *t*-test. The proportion of myeloid was the combined effect of monocyte, macrophage, and dendritic cell types, and T cell was the combined effect of Naïve-like, Treg, transitional, cytotoxic, and proliferating T cell types. **(C)** Side-by-side boxplot indicated the correlation (top) and RMSE (bottom) between the estimated and true cellular proportions, respectively, using BC-specific RGEP and HNSCC-RGEP (derived from HNSCC scRNA-seq data) (14) based on Bassez et al.’s dataset (20). *P*-value, Student’s *t*-test. **(D)** Using the tumor purity of TCGA-BRCA patients estimated by ESTIMATE as the gold standard, scatter-plot showed the degree of consistency of the malignant proportion estimated using BC-specific RGEP (Left) and HNSCC-RGEP (14). (Right) with the gold standard purity. Each dot represents one sample and *r* denotes Pearson’s correlation coefficient. *P-*value, Student’s *t*-test. **(E)** Box plots showed the proportional distribution of fibroblast, immune cells and malignant cells, where the proportion of immune cells was the combined effect of B, mast, monocyte, macrophage, dendritic, pDC, neutrophils, NK, naïve-like, Treg, transitional, cytotoxic and proliferating T cells. Each box shows the median and interquartile range (IQR 25th–75th percentiles), whiskers indicate the highest and lowest value within 1.5 times the IQR and outliers are marked as dots. **(F)** Comparison of the proportions of fibroblasts, immune and malignant cell types in different cancer subtypes. Each box shows the median and interquartile range (IQR 25th–75th percentiles), whiskers indicate the highest and lowest value within 1.5 times the IQR and outliers are marked as dots. Wilcoxon rank-sum test was used for statistical analysis (****p < 0.0001). **(G)** Comparison of the proportions of major immune cell types between primary tumor and solid tissue normal samples. Each box shows the median and interquartile range (IQR 25th–75th percentiles), whiskers indicate the highest and lowest value within 1.5 times the IQR and outliers are marked as dots. Wilcoxon rank-sum test was used for statistical analysis (ns, “no significance”, **p < 0.01, ****p < 0.0001). **(H)** ROC curve measuring the ability to distinguish ER+ and ER- of BRCA samples using cellular proportions estimated by BC-specific RGEP and non-BC-specific RGEPs in combination with the LinDeconSeq deconvolution method (8).

To evaluate the prediction performance of BC-specific RGEP, we first deconvoluted the simulated BC bulk gene expression samples using LinDeconSeq (8), and observed very high consistency (*r* = 1, *P*-value < 1 × 10^-30^) (**Figure 2A**, see *Materials and Methods*). To obtain realistic datasets, we extracted BC scRNA-Seq data (40 BC patients, seven broad cell types, and their proportions/patient were known in advance) from a previously published study (20) (Bassez et al.’s data, see **Supplementary Table S1**). Here again, the deconvolution showed a significantly high correlation (*r* = 0.91, *P-value* < 2.1 × 10^-19^) between predicted and the true proportions (**Figure 2B**). As a proof of principle, we tested another RGEP (called “HNSCC-RGEP”) (14) generated from head and neck squamous cell carcinoma (HNSCC) and found that our BC-specific RGEP made the predictions closer to the true proportions (i.e., higher correlation and lower RMSE) (**Figure 2C**). A similar trend was also observed in the GSE75688 scRNA-Seq dataset (27) (**Supplementary Figures 2A, B** and **Supplementary Table S1**). These results indicate that our BC-specific RGEP can accurately predict the cellular compositions in BC-TME, and with better predictive performance compared to other non-specific references (even generated from different tissues like HNSCC).

We next assess the performance of BC-specific RGEP on traditional bulk transcriptome sequencing data (i.e. bulk RNA-seq data) by determining the proportions of 15 reference cell types in each sample. Here again, we used ESTIMATE (25) to determine the tumor purity, and found that prediction based on BC-specific RGEP showed a higher correlation compared to the one based on HNSCC-RGEP (**Figure 2D**). We also collected the malignant purity of TCGA-BRCA samples predicted by other tools, including ABSOLUTE, LUMP, IHC and CPE algorithms from the literature of Dvir Aran et al. (37). When comparing all of them, our predictions also showed a good/favorable performance (**Supplementary Figures 2C–G**). We also aggregated the predicted proportions of 15 cell types in TCGA-BRCA samples as fibroblasts, immune cells, and malignant cells and observed that the proportion of malignant cells was the highest whereas fibroblasts appeared to be the lowest (**Figure 2E**). The proportions of immune, fibroblast and malignant cells showed significant differences in the PAM50 subtypes of BC, indicating different degrees of infiltration (**Figure 2F**). Moreover, the Normal-like samples showed the highest percentage of fibroblasts, and Luminal B had the lowest number of fibroblasts. Basal-like tumors displayed the highest degree of immune cell infiltration, followed by Her2 tumors, while Luminal A tumors showed the lowest. To avoid the effect of covariance on the deconvolution, we aggregated 13 immune cell types of BC-specific RGEP into seven major lineages (B cells, mast, myeloid, neutrophil, NK, pDC, and T cells) and found that the proportions of B cells, myeloid, NK, and T-cells differed significantly between BC primary tumor and the normal tissue. These observations were consistent with previous studies (**Figures 2E–G**) (19, 38, 39).

To more systematically assess the BC-specific RGEP, we collected three additional non-BC-specific RGEPs, namely LM22 (9), immunoStates (12), and ABIS (13), and used them with LindeconSeq to predict the cellular compositions of TCGA-cohort, respectively. To compare the accuracy and reliability of BC-specific RGEP with non-specific RGEPs for predicting cellular proportions, area under the curve (AUC) index was employed. Specifically, an SVM classifier was used to distinguish ER+ and ER- of BC patients based on the predicted cellular proportions by each RGEP, and then an AUC index was calculated (see *Materials and Methods*). Higher AUC indicates better classification ability, which suggests that this RGEP-predicted cellular composition is more capable of characterizing the intrinsic heterogeneity of BC patients with different subtypes. The result demonstrated that our BC-specific RGEP had the highest AUC (0.89) value (**Figure 2H**), suggesting that it is superior in characterizing the intrinsic heterogeneity of BC patients with different subtypes.

### 3.3 Construction of Functional Gene Set-Based Prognostic Model

**Figure 3:**
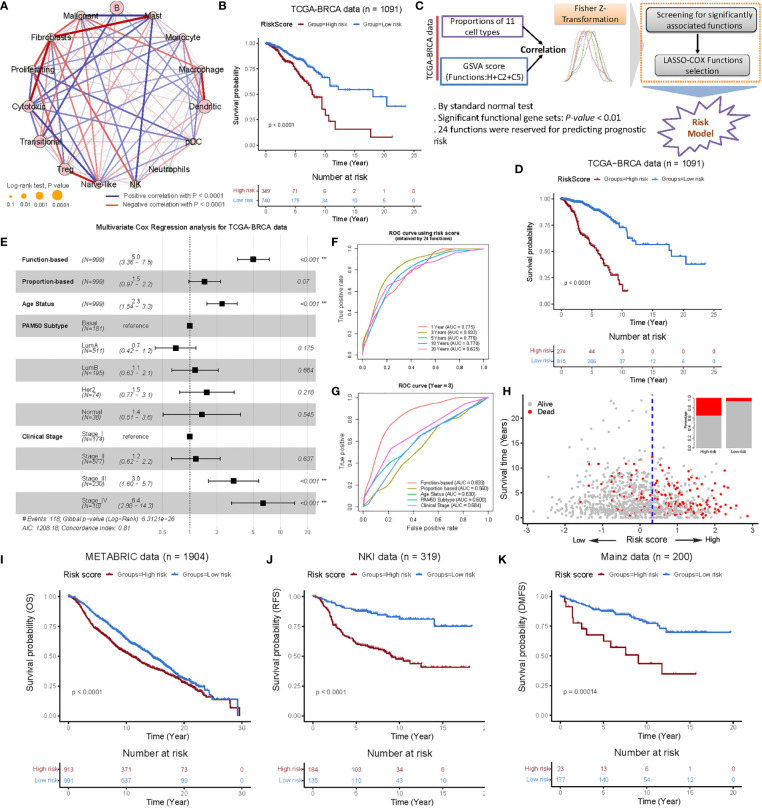
Construction and validation of the functional gene set-based prognostic model in the BRCA cohorts. **(A)** Cellular interaction of the TME cell types. The size and filled color of each circle represent the prognosis effect of each cell type and were scaled by *P*-value. The lines connecting TME cells represent cellular interactions, where the thickness of the line represents the strength of correlation estimated by Spearman’s correlation analysis. A positive correlation is indicated in red and negative correlation in blue. **(B)** Kaplan-Meier survival curves of overall survival (OS) from the TCGA-BRCA cohort using a prognostic model constructed from the proportion of 11 cell types (obtained by LASSO-COX selections) of BC patients. **(C)** Flow chart of constructing functional gene set-based prognostic mode consisted of three parts. First, correlation analysis was performed for the proportion of 11 cell types and the GSVA (29) score of 9,321 functional gene sets, respectively. Second, Fisher-Z-Transformation converted correlations into almost normally distributions, and significant functions with *P-value* < 0.01 could be retained. Third, LASSO-Cox functions selection was employed and then a functional gene set-based prognostic model was constructed. **(D)** Kaplan-Meier survival curves of OS from the TCGA-BRCA cohort using functional gene set-based prognostic model. **(E)** Multivariate analysis of the clinical characteristics, cellular proportion-based risk score and function-based risk score and functional gene set-based risk score with the OS. Log-rank test, **P < 0.01. **(F)** ROC curves of the functional gene set-based risk score at 1, 3, 5, 10 and 20 years after follow-up. **(G)** ROC curves of the clinical characteristics, cellular proportion-based risk score and functional gene set-based risk score at the year three after follow-up. **(H)** The patterns of the OS and survival status between the high- and low- groups for the TCGA-BRCA cohort. **(I-K)** Kaplan-Meier survival curves of OS, RFS and DMFS of patients in the low- and high-risk groups for the METABRIC (40) **(I)**, NKI (41) **(J)** and Mainz (42) **(K)** datasets, respectively. Relapse-free survival: RFS; Distant recurrence-free survival: DMFS.

After scaling the cellular proportions of TCGA-BRCA cohort, we focused on the TME cell network, mainly to determine the suitability of BC-specific RGEP to the tumor-immune cell interactions, cell lineages, and their effects on overall survival (OS) in BC patients (**Figure 3A**). The analysis showed significant differences (log-rank test, *P-value* < 0.05) in survival between the high and low proportion groups of these cells, with the exception of neutrophils (**Figure 3A**). Subsequently, 11 immune cell types were selected by the LASSO-Cox regression model (with minimized lambda) to build the proportion-based prediction model according to multiple Cox regression (**Supplementary Figures 3A, B**, concordance-index: 0.61). We found that the patients stratified into the high-risk score group had significantly worse overall survival compared to the low-risk score group in the TCGA-BC cohort (log-rank test, *P*-value = 7.45 × 10^-7^, see *Materials and Methods*) (**Figure 3B**). Notably, since the accuracy of deconvolution can be influenced by multiple factors (including data type, e.g., microarray/RNA-seq), the accurate identification of stable signatures holds a great value for predicting the prognosis. Therefore, we specifically used gene set variation analysis (GSVA), which provides an advantage over single samples in order to perform comprehensive pathway-centric analyses in an unsupervised manner. Moreover, this strategy also helps to explore the perturbation of key functional gene sets in different patients for the prognosis prediction.

To investigate the association between these cell types and biological functions, we retrieved the H (Hallmark gene sets), C2 (curated gene sets), and C5 (ontology gene sets) collections from the MSigDB database (28), and estimated the GSVA (29) score for each sample. In this way, we retained the 964 functional gene sets with significant correlations (*P-value* < 0.01) and entered them into the LASSO-Cox regression model for features selection. In the end, we obtained 24 functional gene sets (/pathways) and used them (i.e. “GSVA score” of 24 functions) to construct a prognostic model similar to the proportion-based strategy (**Figures 3C** and **Supplementary Figures 3C–F**, concordance index: 0.782, **Table S4**, see *Materials and Methods*). The overall outcome was consistent in the proportion-based model, except that the differences between high- and low-risk groups (determined by the *surv_cutpoint* function) were more significant for the functional gene set-based prognostic model (**Figure 3D**, log-rank test, *P-value* = 5.9 × 10^-33^). Interestingly, the associations with proliferating T-cells and macrophages were observed predominantly among the 24 functional gene sets (**Supplementary Figure 3D**). In particular, the association of proliferating T-cells with nucleosome localization, spindle checkpoint suggests biological processes in cell proliferation, while the associations with macrophages with MHC protein complexes, negative regulation of T-cell receptor signaling pathways suggest involvement in anti-tumor immunity.

To mention, the multivariate Cox analysis revealed that 24 functions (HR: 5.0, 95CI: 3.36-7.5) and tumor stage IV (HR: 6.4, 95CI: 2.88-14.3) were independent prognostic factors for OS in BC patients and can characterize the prognostic risk better than the proportions of 11 cell types (**Figure 3E**). In addition, the area under the curve (AUC) predictive value for the functional gene set-based model showed the highest survival rate by 3 years (**Figure 3F**). As compared to the other clinical characteristics and proportions of 11 cell types, the functional gene set-based model revealed the favourable predictive power (**Figure 3G**). Also, we found that the high-risk group had shorter survival times and more deaths (**Figure 3H**). We additionally tested nine microarray expression datasets (see **Supplementary Table S1**) and observed the significant differences between high- and low-risk groups in these validation cohorts (**Figures 3I–K** and **Supplementary Figures 4A–F**, log-rank test, *P-value* < 0.05). Overall, the analysis in multiple test cohorts suggests that our functional gene set-based prognostic model can clearly define the intrinsic characteristics of BC patients’ prognosis.

### 3.4 Clinical and Biological Characteristics of High- and Low-Risk Groups Depicted by the Functional Gene Set-Based Prognostic Model

**Figure 4:**
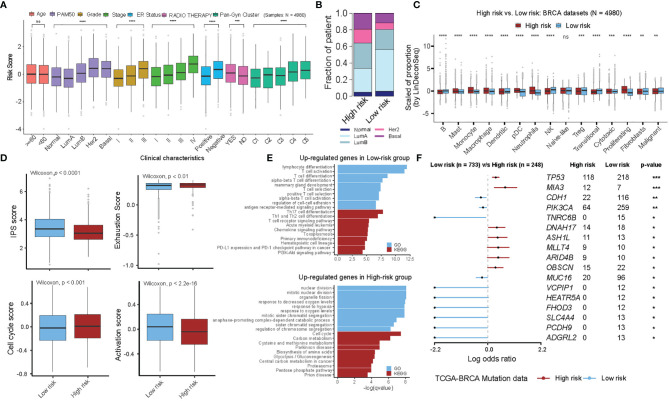
Multi-perspective bioinformatics analysis of clinical and biological characteristics of high- and low-risk groups. **(A)** Stratified analysis of clinical characteristics for the risk score of the functional gene set-based prognostic model in ten BRCA cohorts. Each box shows the median and interquartile range (IQR 25th–75th percentiles), whiskers indicate the highest and lowest value within 1.5 times the IQR and outliers are marked as dots. The dots represent scaled risk score values. Wilcoxon rank-sum test was used for statistical analysis (ns, “no significance”, *p < 0.05, **p < 0.01, ***p < 0.001, **** p < 0.0001). **(B)** The fraction of patients with PAM50 subtypes in the high- and low-risk groups. **(C)** The proportion of TME cells in high- and low-risk groups. Each box shows the median and interquartile range (IQR 25th–75th percentiles), whiskers indicate the highest and lowest value within 1.5 times the IQR and outliers are marked as dots. The dots represent the scaled fraction values of TME cells. Wilcoxon rank-sum test was used for statistical analysis (ns, “no significance”, *p < 0.05, **p < 0.01, ***p < 0.001, **** p < 0.0001). **(D)** The relative distribution of immune signature gene scores was compared between high- and low-risk groups in ten BRCA cohorts. (Left-top) IPS score, (Left-bottom) Cell-cycle score, (Right-top) Exhaustion score and (Right-bottom) PI3K pathway score. **(E)** GO and KEGG analyses for differentially expressed genes in the high- and low-risk groups. Up-regulated genes in low-risk group (top) and in high-risk group (down) are shown. **(F)** Forest plot showing differentially mutated genes between the high- and low-risk groups. Only genes with more than 10 mutations in the samples in one group were included in the analysis. The statistical difference of the two groups was compared through the Fisher exact test. *P < 0.05; **P < 0.01; ***P < 0.001.

The relationship between prognostic risk score and clinical characteristics was further examined in the entire cohorts (10 BC cohorts, 4980 samples, **Supplementary Table S1**). It was found that the risk scores showed significant differences within the clinical characteristics, however, with the exception for age status (**Figure 4A**, Wilcoxon test, *P-value <*0.05). Of importance, each of the five molecular subtypes of PAM50 showed variations, e.g., Luminal A showed the best prognosis with the lowest risk score, whereas Her2 and Basal types were found to be more aggressive with the highest risk scores (**Figures 4A, B**). We also determined several independent factors and found that, a) histologic grading and pathologic staging of BC showed positive progression of stage and risk score, b) the patients with ER-positive showed a tendency to have a better prognosis (and lower risk) compared to ER-negative patients, c) those with or without radiotherapy showed a significant difference and had a higher risk score in the post-radiotherapy cohort. Since, the cohorts were not matched before and after the radiotherapy, thus the differences between them may vary relative to the treatment response. In addition, Pan-Gyn analysis confirmed that a positive trend increases the risk of C1 to C5 (**Figure 4A**). On the basis of deconvolution using LinDeconSeq (8) and BC-specific RGEP, TME cell infiltration of high- and low-risk groups revealed significant differences except for Naïve-like cells (**Figure 4C**). We also calculated the correlations of risk scores with genes from 24 functional sets based on the TCGA-BRCA cohort and extracted the top 10 genes each with the strongest positive and negative correlations, comparing the expression of these genes in the high- and low-risk groups showed significant differences (**Supplementary Figure 5**, *P*-value < 0.05). As shown in **Supplementary Figure 5**, genes with positive correlation have higher expression in high-risk group; conversely, genes with negative correlation have lower expression in high-risk group.

To further investigate the differences in the transcriptome between high- and low-risk groups, we additionally evaluated the number of parameters related to immune signature using the GSVA method. We observed significant differences between the high- and low-risk groups in the immunophenoscore (IPS) variable, i.e., the high-risk group showed a more severe T-cell exhaustion and cell proliferation activity, indicating a suppressed immune response with a worse prognosis (**Figure 4D**, see *Materials and Methods*). The association of risk scores with the expression of key immune checkpoint genes (including *PD-L1* (*CD247*), *PD1* (*PDCD1*), *LAG3*, and *CTLA4*) were explored and significant negative correlation were found, indicating that BC patients with high-risk scores responded poorly to immune checkpoint blockade therapy (**Supplementary Figure 6**). We further substituted the differentially expressed genes between high- and low-risk groups (see **Supplementary Table S6**) and found that the genes upregulated in the low-risk group were mainly enriched in immune-related categories, such as lymphocyte differentiation and Th17 cell differentiation. On the contrary, genes upregulated in the high-risk group were mainly enriched in the categories related to cell proliferation, such as nuclear division, cell cycle, and response to hypoxia (**Figure 4E** and see **Supplementary Table S7**). Interestingly, we also observed that *TP53*, *MIA3* were frequently mutated genes in the high-risk group, while *CDH1* and *PIK3CA* predominated in the low-risk group (**Figure 4F**). Overall, the high- and low-risk groups represented by the 24-functional gene sets prognostic model showed significant differences in the clinical and transcriptomic characteristics, suggesting that the prognostic model can mirror the BC prognosis.

### 3.5 Functional Gene Set-Based Prognostic Model Serves as a Predictive Parameter With Therapeutic Benefit in BC Cohorts

**Figure 5:**
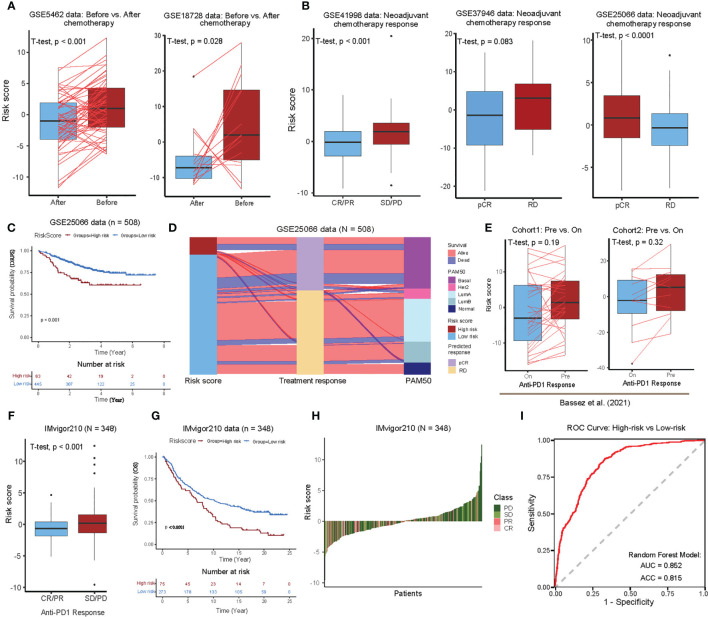
Therapeutic benefit of the 24 functional gene set-based prognostic BC model. **(A)** Pairwise comparison of the risk scores in the patients pre- and post-chemotherapy for the GSE5462 (43) and GSE18728 (44) cohorts. Significance *P*-values were determined by pairwise Student’s *t*-test. **(B)** Boxplot showing the distribution of risk scores for different neoadjuvant chemotherapy response in the GSE41998 (45), GSE37946 (46), and GSE25066 (47) cohorts. Significance *P*-value was determined by Student’s *t*-test. Progressive disease (PD), stable disease (SD), partial response (PR), complete response (CR), pathologic complete response (pCR), and residual disease (RD). **(C)** Kaplan-Meier survival curves of distant recurrence-free survival (DRFS) from the GSE25066 cohort using functional gene set-based prognostic model. **(D)** Alluvial diagram of risk groups with different predicted response (pCR and RD), and molecular subtypes (Normal-like, LumA, LumB, Her2, and Basal). **(E)** Comparisons of risk scores for different status in BC scRNA-seq data provided by Bassez et al. (20). Pairwise comparison of risk scores for the cohort1 (Left) and cohort2 (Right) before and on anti-PD1 treatment. Significance *P*-values was determined by Student’s *t*-test. **(F)** Boxplot showing the distribution of risk scores for different anti–PD1 response in the IMvigor210 cohort. Significance *P*-value was determined by Student’s *t*-test. **(G)** Kaplan-Meier survival curves of OS from the IMvigor210 cohort using functional gene set-based prognostic model. **(H)** Waterfall plot illustrating the distribution of risk scores for patients with different anti-*CTLA4* immunotherapy responses in the IMvigor210 cohort. **(I)** ROC curve of random forest classifier for predicting high and low risk BC patients using 24 functional gene sets.

To investigate whether the risk scores predicted by our functional gene set-based prognostic model can effectively predict the tumor response in BC patients, we performed pairwise comparisons of the risk scores (before and after treatment) with adjuvant chemotherapy, mainly in two BC cohorts (GSE5462 and GSE18728). We found significant differences in the majority of patients who responded with a lower risk score after chemotherapy (**Figure 5A**, see **Supplementary Table S1**). In accordance with patients’ response to neoadjuvant chemotherapy, BC patients (in GSE41998) were further divided into four groups: progressive disease (PD), stable disease (SD), partial response (PR), and complete response (CR). Here, the analysis showed that the risk scores of BC patients with CR/PR were significantly lower than those with SD/PD. The BC patients from the GSE37946 data also showed a significantly lower risk score for pathologic complete response (pCR) compared to the residual disease (RD). To our surprise, the risk score of the pCR cohort was found to be significantly higher compared to RD in the GSE25066 data, which can be partially explained by the intrinsic association between risk score and disease-free survival (DRFS), i.e., high risk favored good prognosis in this particular data set (**Figures 5B, C**). The association between risk score, treatment response and PAM50 subtypes was further investigated, and found that the high-risk group was mainly enriched for Her2 and Basal aggressive subtypes with predominantly pCR status, while the low-risk subgroup was mainly LumA, LumB, and Normal-like with predominantly RD status. This may suggest that the difference in risk between different tumor subtypes is greater than the difference before and after treatment of the consent subtype (**Figure 5D**).

We further investigated whether the risk score could predict immunotherapeutic benefit for BC patients. For this purpose, we used scRNA-seq data from two cohorts consisting of 40 BC patients who received anti-PD1 therapy for approximately 10 days (see **Supplementary Table S1**). The pairwise comparisons of risk scores (before and after immunotherapy treatment) showed low-risk scores after the treatment in both cohorts, however, it was not significant (**Figure 5E**). In the absence of any published datasets of BC patients receiving immunotherapy, we utilized urothelial cancer dataset that received anti-PD-L1 therapy (IMvigor210), in order to test our functional gene set-based prognostic model to classify high- and low-risk groups. The boxplots further showed that the risk scores were significantly low in the patients with complete or partial response (CR/PR) compared to those with stable or progressive disease (SD/PD) (**Figure 5F**). In addition, the Kaplan-Meier curves showed that the patients in the low-risk group had a significantly better prognosis than those in the high-risk group (**Figure 5G**). In the ranking of risk scores from low to high, the low-risk side was enriched with PR/CR patients, whereas the high-risk side was predominated with SD/PD patients (**Figure 5H**). Overall, these analyses suggest that the risk scores calculated by our BC functional gene set-based prognostic model perform well for stratifying response to the immunotherapy.

In order to build the classifier that could predict the high- and low-risk group for BC patients, we applied the random forest algorithm (R package *randomForest*, version 4.6) using the GSVA scores of 24 functional gene sets as features in the training cohorts (ten BC cohorts, 80% for training and the remaining 20% for testing) (see *Materials and Methods*, see **Supplementary Table S1**). And we found the overall accuracy and AUC of the test cohorts as 81.5% and 0.852, respectively, showing a favorable predictive power (**Figure 5I**). Of note, interleukin 21-mediated signaling and protein localization in the nucleoplasm emerged as the most important features in our analysis (**Supplementary Figure 7**).

### 3.6 Extending the Functional Gene Sets-Based Prognostic Model of BC to Pan-Cancer

**Figure 6:**
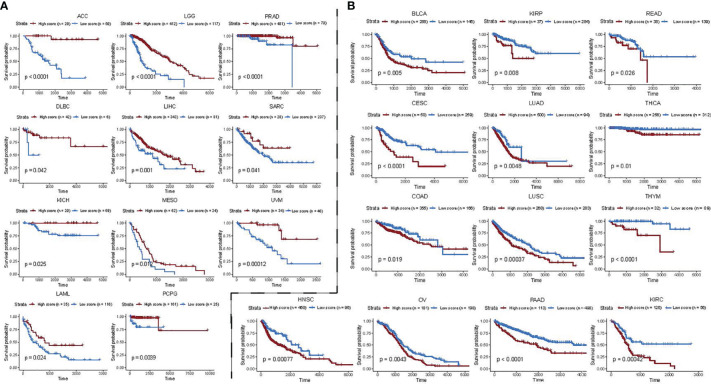
Functional gene set-based model of BC patients as a prognostic factor for 24 other cancer types. **(A, B)** Impact of risk scores derived from BC functional gene set-based prognostic model on survival of pan-cancer patients. A high score is associated with both worse **(A)** and better **(B)** overall survival. Overall survival of patients with high score was compared with those with low score in a Kaplan–Meier survival curve analysis. Statistical significance was assessed by log-rank test. Only significant cancers with *P*-value < 0.05 were shown.

Next, we investigated whether the association of 24 functional gene sets which we found in BC also applies to other cancers. To achieve this, we used the BC prediction model to calculate risk scores for 32 other cancers in the TCGA database (except BRCA cancers) and used the optimal cut point as an additional parameter to divide patients into two groups per cancer type. Then Kaplan–Meier survival curve analysis was performed between the high- and the low-expression groups. Among 32 cancer types, we found BC functional gene set-based prognostic model was significantly associated with overall survival in 24 cancer types (**Figures 6A, B**). In ACC, LGG, PRAD, DLBC, LIHC, SARC, KICH, MESO, UVM, LAML, and PCPG, the risk score obtained from 24 prognosis-related functional gene sets was observed as a favourable survival factor (**Figure 6A**), while the score was associated with worse survival in BLCA, KIRP, READ, CESC, LUAD, THCA, COAD, LUSC, THYM, HNSC, OV, PAAD, and KIRC (**Figure 6B**).

Taken together, our results suggest that the 24 functional genes closely associated with BC prognosis may have general prognostic significance for all other cancers.

## 4 Discussion

Cancer is a multifactorial disease that combines yet to be known initial causative factors with the dysregulated biological pathways to reshape the genome (48, 49). In fact, cancer heterogeneity is a major challenge in the clinical setup and to some extent contributes to the treatment failure and/or acquires resistance in the cancer patients. It is also now well established that varying combinations of the different cell types and their cross-talk with tumor cells modulate TME, which further complicates the scenario. To address these challenges, advanced methods such as scRNA-seq have taken the central stage. Particularly in BC, a few scRNA-seq studies have been performed to gain better insight into the complex interactions between the immune system and tumor cells (15, 50). This in turn also raises some concerns about how to effectively explore this BC-TME heterogeneity with a direct prognostic/diagnostic clinical application. Since several studies have shown that reference-based deconvolution methods provide an important means to resolve the cellular compositions of bulk samples (5, 6, 8, 9, 11, 51), this prompted us to investigate the heterogeneity of BC-TME and patient prognosis by combining reference expression profiles (RGEPs).

Herein, we constructed a BC-specific RGEP using 15 cell types derived from scRNA-seq data of eight BC patients by considering multiple factors such as tissue and tumor types, disease status, data source (single-cell or sorted bulk data), and signature gene selection (**Figures 1A–D**). By benchmarking different gene expression reference profiles, we showed that the estimation accuracy is ultimately limited by the origin and quality of the RGEPs (**Figures 2A-H**). Moreover, we confirmed that when deconvolution algorithms are combined with scRNA-seq from tumor biopsies, the indication-specific consensus profiles of immune, stromal and malignant cells can be obtained directly from TME. Importantly, we observed that the proportions of both fibroblasts and immune cells estimated by BC-specific RGEP showed significant differences between the molecular subtypes of BC patients (**Figure 2F**), thus validating the direct clinical application of this novel tool. We observed that basal-like and Her2 tumors had the highest median degree of immune cell infiltration, whereas Luminal A tumors showed the lowest. Moreover, these differences profoundly affect the clinical treatment strategy and prognosis of BC patients, as confirmed by univariate Cox regression analysis which was based on the cellular proportions estimated with BC-specific RGEP (**Figure 3A**). Of note, even though we used the deconvolution tools similar to the previously reported non-specific RGEP studies, a slight variation in the outcome of certain variables (e.g., infiltration score of TME and patient prognosis) can be expected due to the additional clinical parameters which we have introduced in our current analysis.

We also evaluated transcriptome sequencing data and found that the cellular proportion-based on our prognostic model can well predict the prognosis of TCGA-BRCA cohorts, and confirming the previous studies (**Figure 3B**) (16, 52). Since the accuracy of deconvolution is affected by many factors (such as data type (microarray/RNA-seq)), we attempted to find stable signatures for predicting the prognosis of BC patients. Therefore, we used gene set variation analysis (GSVA) (29), which works on single samples and allows comprehensive pathway-centric analyses using statistical ranks in an unsupervised manner. Moreover, the correlations between the proportions of 11 cell types and 9,321 functional gene sets were analyzed independently to enhance the analysis. The functional gene sets that were significantly associated with cellular proportions were used in Lasso-Cox regression, and 24 functions were retained for the construction of BC prediction models (**Figures 3C, D**). The validation in ten BC cohorts demonstrated that our functional gene set-based prognostic model has good predictive power (**Figures 3I–K** and **S4A–F**). Despite the selection of features (24 functional gene sets) from different TCGA-BRCA cohorts, it was still possible to visualize the common hallmarks among BC cohorts using GSVA.

Given that prognostic risk scores provide individualized risk estimates for an outcome, the risk scores estimated by our functional gene set-based prognostic model adequately reflected the clinical characteristics of BC patients (**Figure 4A**). Also, when determined by the optimal cut-off point for the risk score, both high- and low-risk groups showed distinct transcriptional characteristics. For instance, the genes that were up-regulated in the low-risk group were mainly enriched in immune-related categories, whereas genes that were up-regulated in the high-risk group were mainly enriched in categories related to cell proliferation (**Figures 4D, E**). Regarding the assessment of patient response to the therapy (chemotherapy and immunotherapy), the obtained risk scores also showed good discrimination between pre- and during/post-treatment (**Figures 5A–H**). Specifically, the selective 24 functions showed good predictive power in discriminating the high- and low-risk samples (**Figure 5I**). We further extended the BC functional gene set-based prognostic model to pan-cancer, and demonstrated the model is also suitable to other 24 cancers types (**Figures 6A, B**). Taken together, the functional gene set-based prognostic model that we have introduced showed a great ability to screen patients based on their therapeutic response. On a broader perspective, we provide a perspective to generate similar models in other cancer types and to identify shared factors that drives cancer heterogeneity.

It is also important to discuss the limitations of this current study, 1) some cell types that are lineage closely in the BC-specific RGEP are highly correlated, which may affect the accuracy of deconvolution, b) similar to other prognostic models, here also the difficulty of using the standardized cut-off for interpreting the risk scores remains. Nevertheless, our analysis showed that our refined BC-specific RGEP reflect the intrinsic expression of cells, and the proposed functional gene set-based prognostic model is a robust one for survival prediction and treatment guidance in BC patients. Thus, its implementation may help in stratifying BC patients to get benefit from adjuvant chemotherapy and cancer immunotherapy. Indeed, the experimental validation of our results may be highly valuable to elucidate the clinical spectrum of BC. On a broader perspective, we provide a perspective to generate similar models in other cancer types to identify shared factors that drives cancer heterogeneity.

## Data Availability Statement

The datasets presented in this study can be found in online repositories. The names of the repository/repositories and accession number(s) can be found in the article/**Supplementary Material**.

## Author Contributions

HDL and XS designed the study. HML coded the algorithms. HML, HDL, and AS wrote and revised the manuscript. HML, YTH, and WLM did data analysis. HDL, KL, ZG and XS provided interpretation and discussion. All authors contributed to the article and approved the submitted version.

## Funding

This work was supported by the National Natural Science Foundation of China (No. 61972084), the Key Research & Development Program of Jiangsu Province (BE2016002-3), “the Open Research Fund of State Key Laboratory of Bioelectronics, Southeast University” and the project of Southeast University (No. 3207032101F, and No. 3207032101C3).

## Conflict of Interest

The authors declare that the research was conducted in the absence of any commercial or financial relationships that could be construed as a potential conflict of interest.

## Publisher’s Note

All claims expressed in this article are solely those of the authors and do not necessarily represent those of their affiliated organizations, or those of the publisher, the editors and the reviewers. Any product that may be evaluated in this article, or claim that may be made by its manufacturer, is not guaranteed or endorsed by the publisher.

## Supplementary Material

Supplementary Table S1 Datasets and their application in this study.

Supplementary Table S7 Gene ontology and KEGG pathway enrichment analysis.
