# Supplementary material for: From Cellular Infiltration Assessment to a Functional Gene Set-Based Prognostic Model for Breast Cancer
Source: Front Immunol. 2021 Oct 4;12:751530. doi: 10.3389/fimmu.2021.751530 (PMC8529968; doi:10.3389/fimmu.2021.751530)
Supplement: Supplementary Table S1 — Datasets and their application in this study. [file DataSheet_1.zip › SuppFiles/Frontiers_BC_Supplementary_Materials.pdf]

Supplementary Material

1 Supplementary Figures

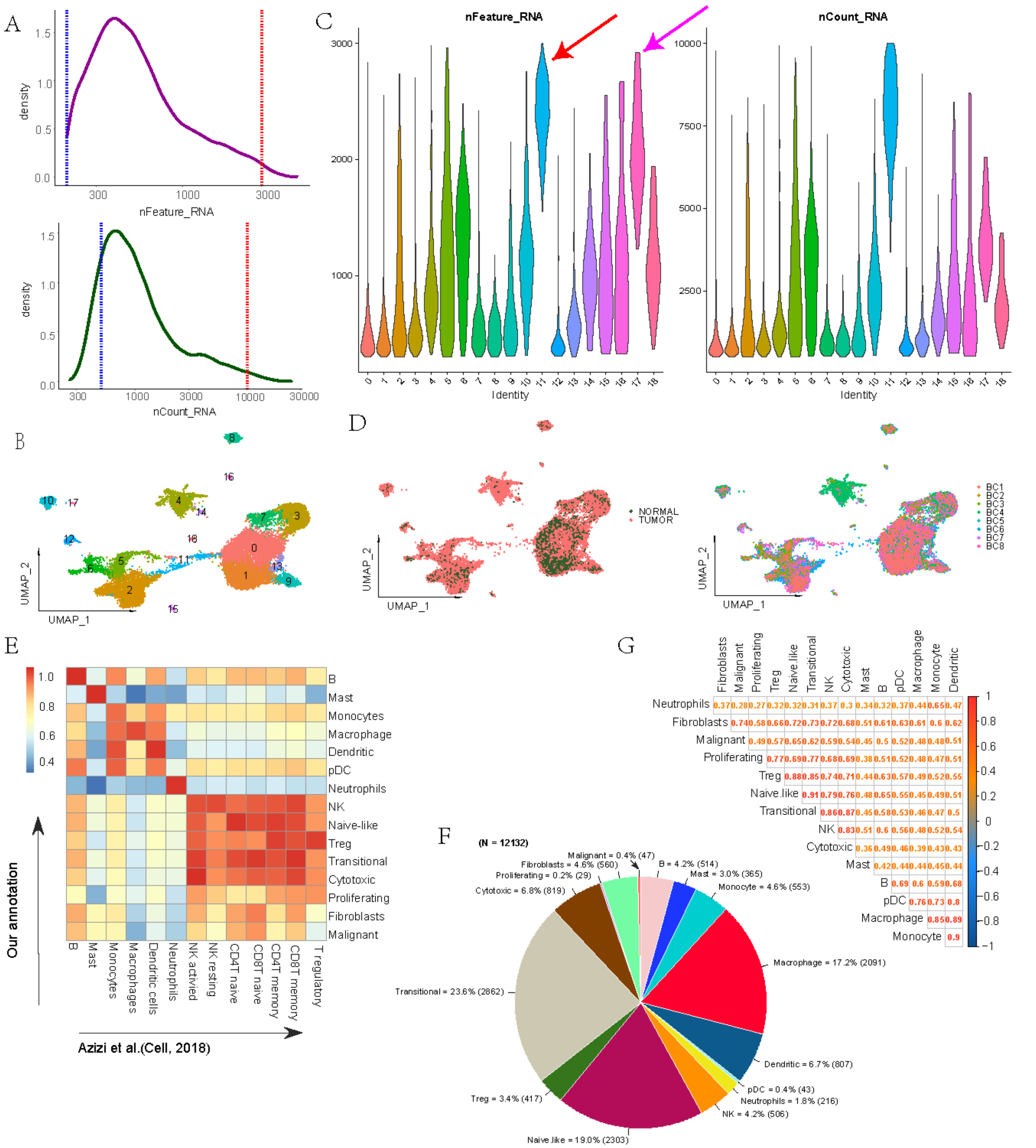

Supplementary Figure 1. Preprocessing, clustering and statistics of scRNA-seq data of eight BC patients. Density-plot showed the distribution of scRNA-seq data of eight BC patients from two different aspects, including expressed genes per cell (Top) and total UMIs per cell (Bottom). The blue and red dotted lines indicate that cells below or above the corresponding threshold were filtered out. (A) UMAP showed 19 clusters of single cells with a resolution of 0.5 after Harmony [1] integrated. The clusters were marked by different colors. Single cells were shown in dots. (B) Violin-plot depicting the number of gene and number of UMI in each cluster. Arrows with lines indicated outlier clusters. (C) UMAP showed the distribution of single cells after CCA integrated. (Left)

Normal vs. Tumor (Right) Patients. (D) Correlation between aggregated expression profiles from cell-type clusters determined by us and previous study [2]. (E) Pie-plot showed the percentage and count of cells in each cell type. (F) The Correlation plot showed Spearman's Rho between cell types in the derived signature profile.

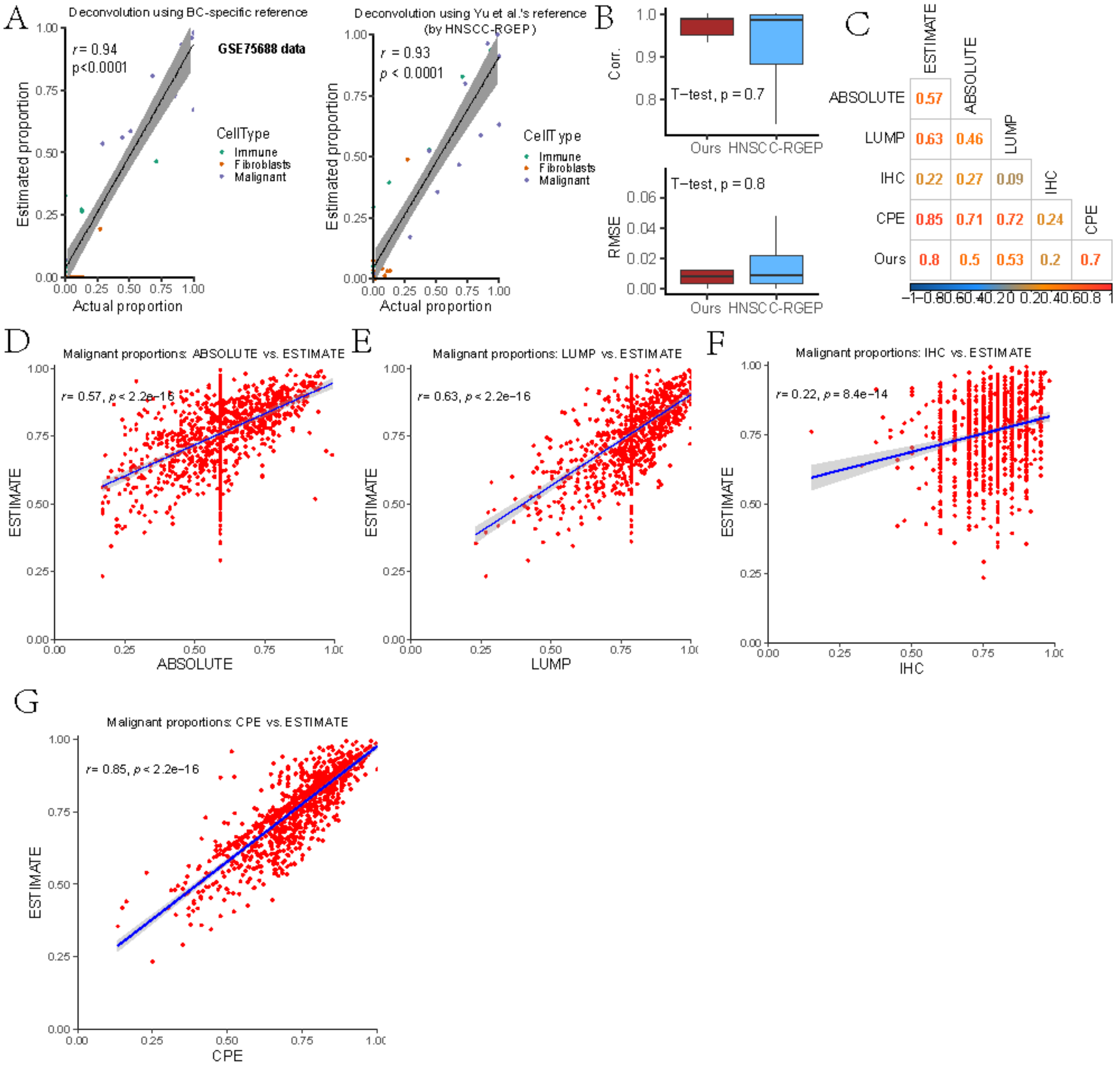

Supplementary Figure 2. Performance comparison with other non-specific RGEP and tumour purity prediction tools. (A) Scatter-plot of the estimated and true cellular proportions for the GSE75688 scRNA-seq data [3]. (Left) Our BC-specific RGEP (Right) HNSCC-RGEP (derived from HNSCC scRNA-seq data by Yu et al.) [4]. Each dot represents one patient and *r* denotes the Pearson's correlation coefficient. P value, Student's t-test. The proportion of Immune was the combined effect of B, Mast, Monocyte, Macrophage, Dendritic cells, pDC, Neutrophils, NK, Naïve-like, Treg, Transitional, Cytotoxic, and Proliferating T cell types. (B) Side-by-side boxplot indicated the Pearson's correlation coefficient (Top) and RMSE (Bottom) between estimated and true cell proportions, respectively, based on the GSE75688 dataset using BC-specific RGEP and HNSCC-RGEP. *P*-value, Student's t-test. (C) Pairwise correlations of predicted malignant proportion between different tools in TCGA-BRCA patients/samples. (D-G) Using the tumour purity of TCGA-BRCA patients estimated by ESTIMATE [5] as the gold standard, scatter-plot showed the degreed of consistency between the malignant proportions estimated using different tools and the gold standard purity. Each dot represents one sample and r denotes Pearson's correlation coefficient. *P*-value, Student's t-test.

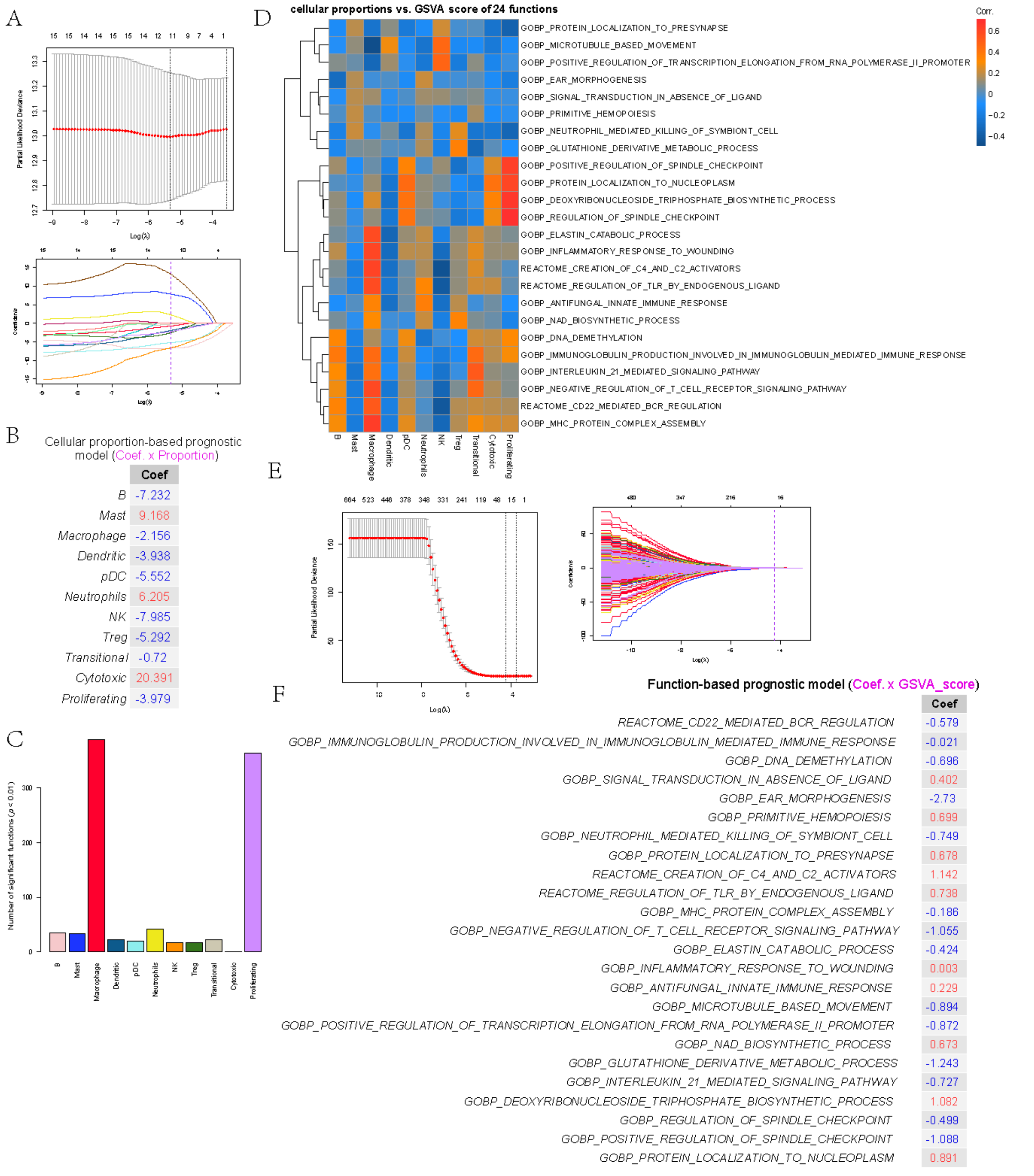

Supplementary Figure 3. Proportion-based and functional gene set-based prognostic model. (A) Partial likelihood deviance revealed by the LASSO regression model in the 10-fold cross validation. The vertical dotted lines were drawn at the optimal values by using the minimum and 1-SE criteria (Top). LASSO coefficient profiles of 15 cell types in the 10-fold cross validation. The vertical dotted lines were drawn at the optimal values by using the minimum criteria and 1-SE criteria, colored according to cell types (Bottom). (B) Cellular proportion-based prognostic model. The risk score of a sample is a linear combination of the selected cellular proportions, where the coefficients were

derived from a multivariate Cox model based on the TCGA-BRCA cohort. (C) Number of functions significantly correlated with each cell type. Colored according to cell types. (D) Heat-map plot showed the Pearson's correlation coefficients between proportions of selected cell types and GSVA [6] scores of 24 functional gene sets, which were derived from the set of significant functions by the Lasso-Cox feature selection model. (E) Partial likelihood deviance revealed by the LASSO regression model in the 10-fold cross validation. The vertical dotted lines were drawn at the optimal values by using the minimum and 1-SE criteria (Left). LASSO coefficient profiles of 964 significant gene sets in the 10-fold cross validation. The vertical dotted lines were drawn at the optimal values by using the minimum criteria and 1-SE criteria, colored according to cell types (Right). (F) Functional gene set-based prognostic model. The risk score of a sample is a linear combination of the GSVA scores for 24 functional gene sets, where the coefficients were derived from a multivariate Cox model based on the TCGA-BRCA cohort.

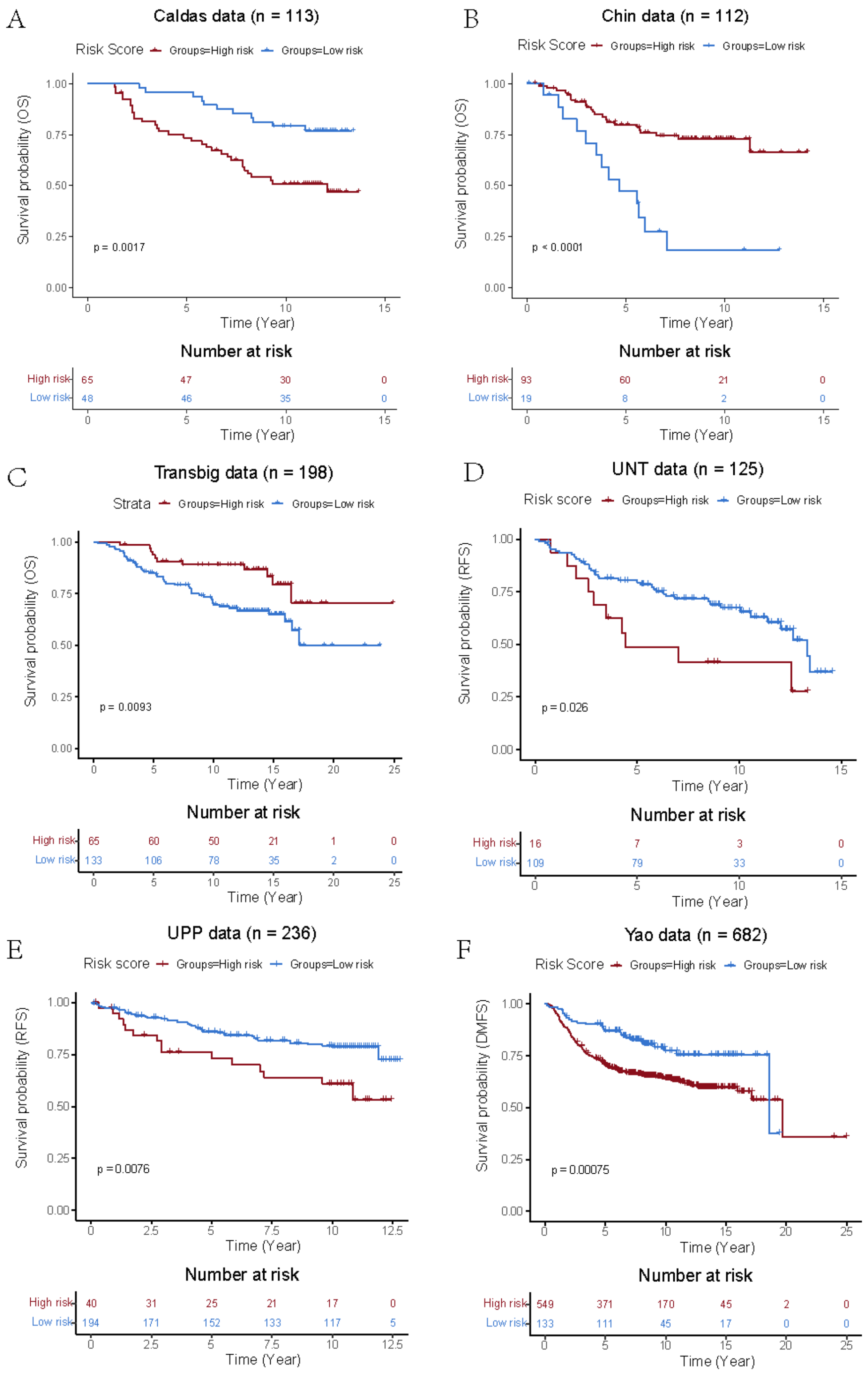

Supplementary Figure 4. Performance of the functional gene set-based prognostic model was validated on six other BC cohorts. (A-F) Kaplan-Meier survival curves between the high- and low-groups for six BC cohorts using our function-based prognostic model. (A) Caldas data (OS), (B) Chin data (OS), (C) Transbig data (OS), (D) UNT data (RFS), (E) UPP data (RFS), and (F) Yao data (DMFS). Relapse-free survival: RFS; Distant recurrence-free survival: DMFS.

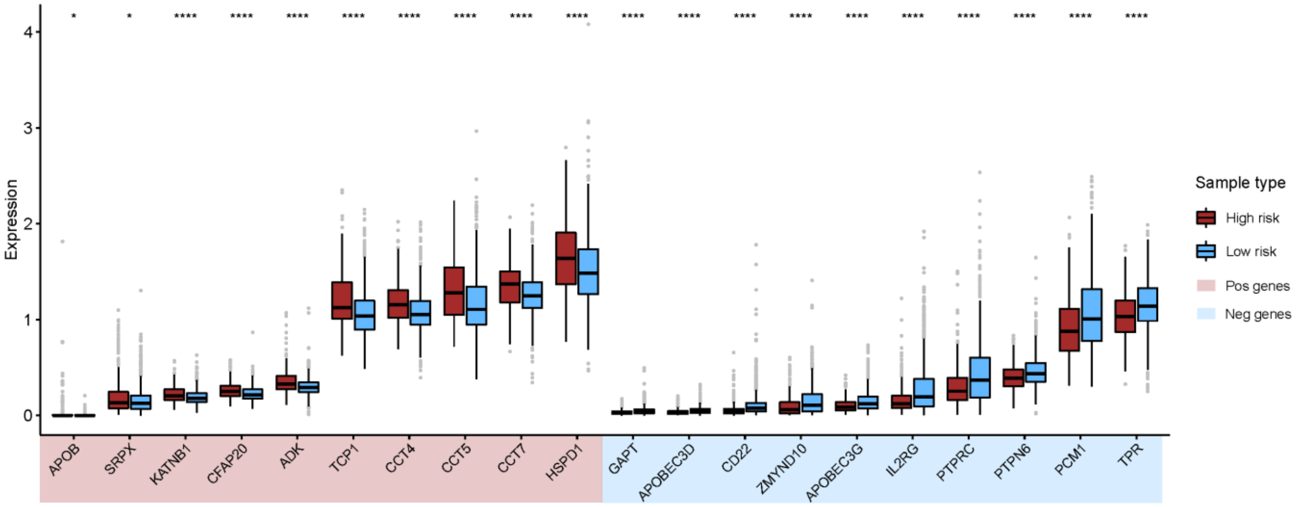

Supplementary Figure 5. Comparison of the expression of genes highly associated with risk scores in high- and low-risk groups. The dots represent normalized expression values. Wilcoxon rank-sum test was used for statistical analysis (*$p < 0.05$, **$p < 0.01$, ***$p < 0.001$).

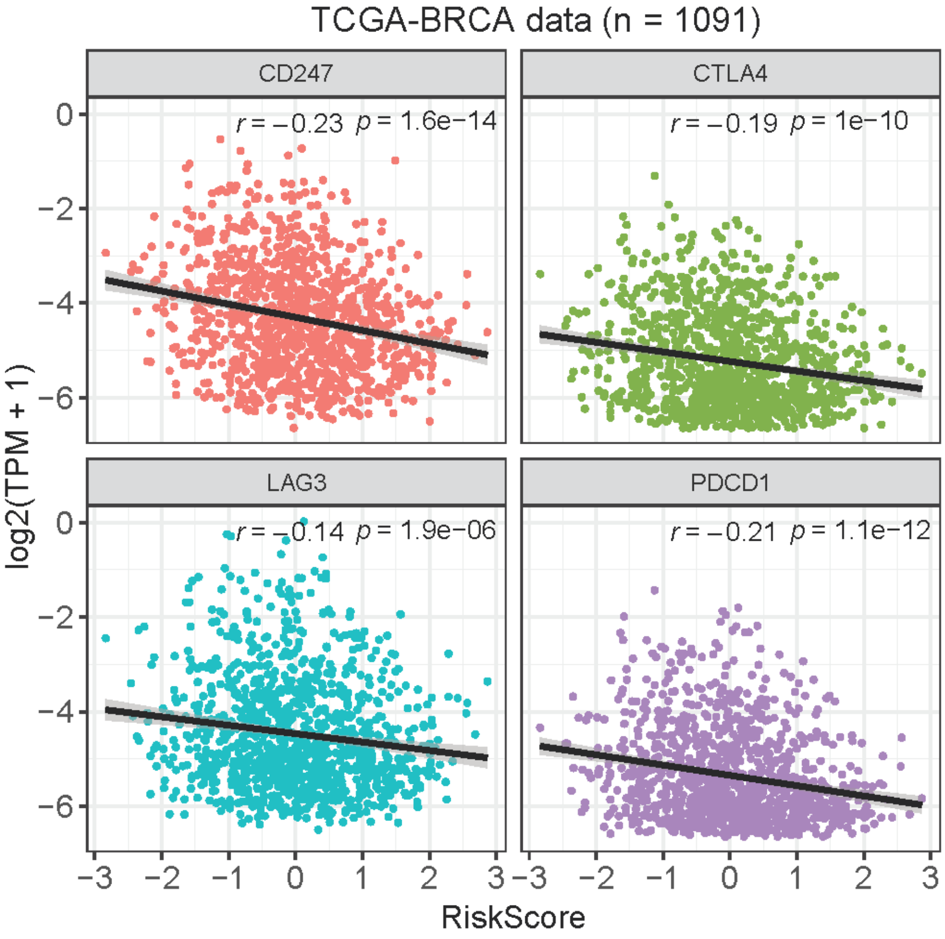

Supplementary Figure 6. Correlations between risk score and expression of key immune checkpoint genes. (Left-top) *CD247* (alias: *PD-L1*); (Left-bottom) *LAG3*; (Right-top) *CTLA4*; and (Right-bottom) *PDCD1* (alias: *PD1*).

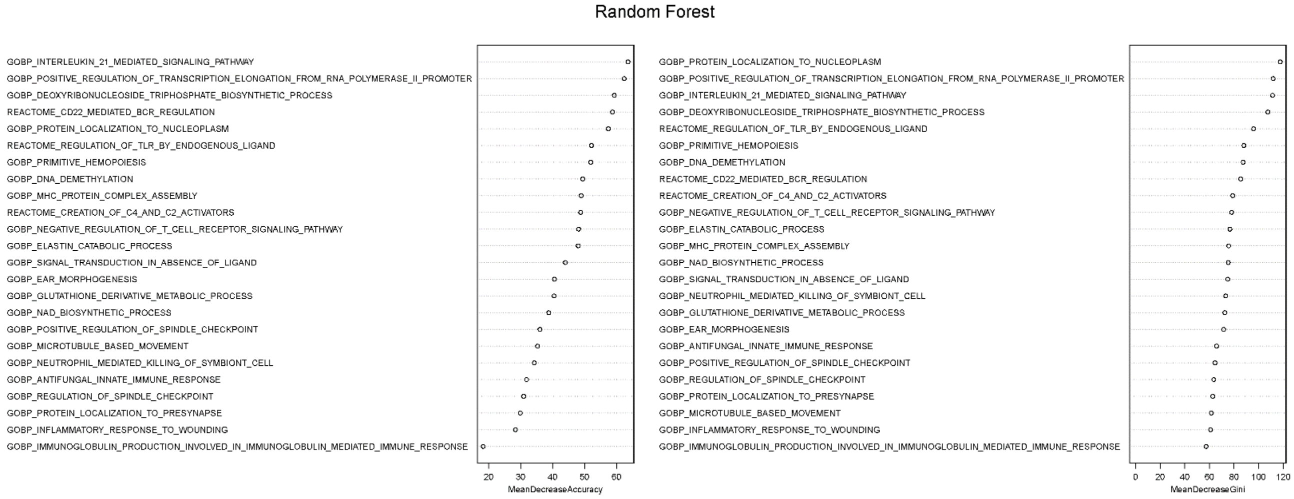

Supplementary Figure 7. Feature importance ranking by a random forest algorithm. The importance of selected 24 functional gene sets in risk model were ranked by a random forest algorithm, ordered by mean decrease accuracy (left) or mean decrease Gini (right) separately.

Reference

1. Korsunsky I, Millard N, Fan J, Slowikowski K, Zhang F, Wei K, Baglaenko Y, Brenner M, Loh PR, Raychaudhuri S: **Fast, sensitive and accurate integration of single-cell data with Harmony**. *Nat Methods* 2019, **16**(12):1289-1296.
2. Azizi E, Carr AJ, Plitas G, Cornish AE, Konopacki C, Prabhakaran S, Nainys J, Wu K, Kiseliovas V, Setty M *et al*: **Single-Cell Map of Diverse Immune Phenotypes in the Breast Tumor Microenvironment**. *Cell* 2018, **174**(5):1293-1308 e1236.
3. Chung W, Eum HH, Lee HO, Lee KM, Lee HB, Kim KT, Ryu HS, Kim S, Lee JE, Park YH *et al*: **Single-cell RNA-seq enables comprehensive tumour and immune cell profiling in primary breast cancer**. *Nature communications* 2017, **8**:15081.
4. Yu X, Chen YA, Conejo-Garcia JR, Chung CH, Wang X: **Estimation of immune cell content in tumor using single-cell RNA-seq reference data**. *BMC Cancer* 2019, **19**(1):715.
5. Yoshihara K, Shahmoradgoli M, Martinez E, Vegesna R, Kim H, Torres-Garcia W, Trevino V, Shen H, Laird PW, Levine DA *et al*: **Inferring tumour purity and stromal and immune cell admixture from expression data**. *Nature communications* 2013, **4**:2612.
6. Hänzelmann S, Castelo R, Guinney JJBb: **GSVA: gene set variation analysis for microarray and RNA-seq data**. 2013, **14**(1):7.
